# Occupational Asbestos Exposure and Incidence of Colon and Rectal Cancers in French Men: The Asbestos-Related Diseases Cohort (ARDCo-Nut)

**DOI:** 10.1289/EHP153

**Published:** 2016-08-12

**Authors:** Christophe Paris, Isabelle Thaon, Fabrice Hérin, Benedicte Clin, Aude Lacourt, Amandine Luc, Gaelle Coureau, Patrick Brochard, Soizick Chamming’s, Antoine Gislard, Pilar Galan, Serge Hercberg, Pascal Wild, Jean-Claude Pairon, Pascal Andujar

**Affiliations:** 1 EA7298 INGRES (Interactions gènes-risques environnementaux et effets sur la santé), Faculté de Médecine, Université de Lorraine, Vandoeuvre Les Nancy, France; 2 CHU (Centre Hospitalier Universitaire) Nancy, Vandoeuvre Les Nancy, France; 3 UMR (Unité Mixte de Recherche) 1027, Université de Toulouse, Toulouse, France; 4 CHU Toulouse, Toulouse, France; 5 INSERM (Institut national de la santé et de la recherche médicale) U1086, Cancers et Populations, Caen, France; 6 Université Segalen, Bordeaux, France; 7 INSERM 1219, EPICENE (Epidémiologie du cancer et expositions environnementales), Bordeaux, France; 8 CHU Bordeaux, Bordeaux, France; 9 IIMTPIF (Institut Interuniversitaire de Médecine du Travail de Paris Ile de France), Créteil, France; 10 CHU Rouen, Service de Pathologie professionnelle, Rouen, France; 11 INSERM U1153, Nutritional Epidemiology Research Team (EREN), Bobigny, France; 12 INRS (Institut national de recherche et de sécurité), Direction scientifique, Vandoeuvre Les Nancy, France; 13 Institut Santé Travail Paris-Est, Université Paris-Est, Créteil, France; 14 Service de Pneumologie et Pathologie Professionnelle, DHU A-TVB (Ageing-Thorax-Vessels-Blood), CHI Créteil (Centre Hospitalier Intercommunal de Créteil), Créteil, France; 15 INSERM U955, Equipe 4, Créteil, France; 16 Faculté de Médecine, Université Paris-Est Créteil, Créteil, France

## Abstract

**Background:**

The relationships between asbestos exposure and colorectal cancer remain controversial.

**Objectives:**

We examined the association between asbestos exposure and colorectal cancer incidence.

**Methods:**

Volunteer retired workers previously exposed to asbestos were invited to participate in the French ARDCo screening program between 2003 and 2005. Additional data on risk factors for colorectal cancer were collected from the ARDCo-Nut subsample of 3,769 participants in 2011. Cases of colon and rectal cancer were ascertained each year through 2014 based on eligibility for free medical care following a cancer diagnosis. Survival regression based on the Cox model was used to estimate the relative risk of colon and rectal cancer separately, in relation to the time since first exposure (TSFE) and cumulative exposure index (CEI) to asbestos, and with adjustment for smoking in the overall cohort and for smoking, and certain risk factors for these cancers in the ARDCo-Nut subsample.

**Results:**

Mean follow-up was 10.2 years among 14,515 men, including 181 colon cancer and 62 rectal cancer cases (41 and 17, respectively, in the ARDCo-Nut subsample). In the overall cohort, after adjusting for smoking, colon cancer was significantly associated with cumulative exposure (HR = 1.14; 95% CI: 1.04, 1.26 for a 1-unit increase in ln-CEI) and ≥ 20–40 years since first exposure (HR = 4.67; 95% CI: 1.92, 11.46 vs. 0–20 years TSFE), and inversely associated with 60 years TSFE (HR = 0.26; 95% CI: 0.10, 0.70). Although rectal cancer was also associated with TSFE 20–40 years (HR = 4.57; 95% CI: 1.14, 18.27), it was not associated with ln-CEI, but these findings must be interpreted cautiously due to the small number of cases.

**Conclusions:**

Our findings provide support for an association between occupational exposure to asbestos and colon cancer incidence in men.

**Citation:**

Paris C, Thaon I, Hérin F, Clin B, Lacourt A, Luc A, Coureau G, Brochard P, Chamming’s S, Gislard A, Galan P, Hercberg S, Wild P, Pairon JC, Andujar P. 2017. Occupational asbestos exposure and incidence of colon and rectal cancers in French men: the Asbestos-Related Diseases Cohort (ARDCo-Nut). Environ Health Perspect 125:409–415; http://dx.doi.org/10.1289/EHP153

## Introduction

Asbestos exposure is associated with several malignancies including lung cancer, mesothelioma, and more recently, ovarian and laryngeal cancers (IARC 2012). However, the role of asbestos in the pathogenesis of digestive cancers, particularly colorectal cancer, remains controversial. Since 1964, when Selikoff et al. (1964) reported a positive association among American insulators for colorectal cancer, numerous studies on this association have been published. In 1994, two meta-analyses reported an overall significant association between asbestos and colorectal cancer, but failed to demonstrate the existence of a dose–response relationship (Gamble 1994; Homa et al. 1994). The most recent IARC review noted that contributors were “evenly divided as to whether the evidence was strong enough to warrant classification as sufficient” for asbestos and colorectal cancer (IARC 2012) given that some studies reported significant positive associations (Aliyu et al. 2005; Berry et al. 2000; Clin et al. 2011; Germani et al. 1999; Kang et al. 1997), while others did not (Battista et al. 1999; Clin et al. 2009; Dement et al. 1994; Ferrante et al. 2007; Giaroli et al. 1994; Hein et al. 2007; Levin et al. 1998; Rösler et al. 1994). Studies of asbestos and colorectal cancer published after the 2012 IARC review include two that reported an association (Lin et al. 2014; Offermans et al. 2014) and two that reported no association (Loomis et al. 2009; Wang et al. 2013a). Consequently, the putative association between colorectal cancer and asbestos exposure remains controversial.

A large-scale CT-scan screening program for asbestos-related diseases was initiated in four regions of France in 2003 following a national consensus conference on the clinical surveillance strategy for former asbestos workers. Male and female volunteers who were retired or unemployed workers and who were previously occupationally exposed to asbestos were eligible to participate in the Asbestos-related Diseases Cohort (ARDCo). We have previously reported that the prevalence of pleural plaques and asbestosis ranges between 7.3% and 36.8% and between 1.8% and 13.7%, respectively, according to occupation, industrial sector, and the level of cumulative asbestos exposure in this population (Paris et al. 2009). In the same article, we also reported that the cumulative exposure index (CEI) and time since first exposure (TSFE), but not the duration of the exposure, were significantly associated with the prevalence of pleural plaques.

These participants have subsequently been followed each year, and the incidence or mortality of various cancers has been recorded during follow-up of the cohort (Pairon et al. 2013, 2014). The present study was designed to examine the putative association between asbestos exposure and risk of colorectal cancer in a 10-year follow-up study of formerly asbestos-exposed workers.

## Methods

### Inclusion in the Overall Study Population

A screening program for asbestos-related diseases was organized between October 2003 and December 2005 in four regions of France (Haute-Normandie, Basse-Normandie, Aquitaine, and Rhône-Alpes). Volunteer participants involved in this program were enrolled from various industrial sectors such as iron and steel manufacturing, the construction sector, cargo handling, metalworking, or ship repair (Paris et al. 2009). Retired or unemployed volunteers were invited to participate in the program in various ways according to the region (e.g., letters, radio announcements, television, and newspaper advertisements were used to target age groups < 60 to ≥ 75 years old and previous type of job, such as trade union). After hygienists confirmed previous occupational exposure to asbestos based on a questionnaire, participants received a free medical examination, including a chest computed tomography (CT) scan and pulmonary function tests after enrollment (Paris et al. 2009; Clin et al. 2011; Ameille et al. 2010).

The study was approved by the hospital ethics committee [Comité Consultatif de Protection des Personnes dans la Recherche Biomédicale (CCPPRB) and Comité de Protection des Personnes (CPP)]. All participants received information about the study and gave their written informed consent.

### Asbestos Exposure and Tobacco Consumption

Participants provided a complete work history, and an industrial hygienist independently coded the dates and duration (years) of exposure for each job associated with asbestos exposure. In addition, each job was classified with regard to the intensity of exposure, and a CEI in exposure units × years was derived as the sum of the duration × intensity weighting factor (low or passive exposure: 0.01; intermediate: 0.1; high intermediate: 1.0; high: 10.0) for each asbestos-exposed job. Measurements of atmospheric asbestos concentrations were not available (Pairon et al. 2013). Smoking status was recorded at enrollment (never smoker, former smoker for > 1 year, current smoker including former smoker having stopped smoking for < 1 year). We also included a missing data category for smoking.

### Data Collection of Risk Factors for Digestive Cancers: the ARDCo-Nut Sample

In 2011, a new questionnaire was mailed to all male participants who were not known to be deceased. This questionnaire was mainly designed to assess risk factors for digestive cancers, namely body mass index (BMI), exercise, familial adenomatous polyposis (FAP), and a family history of colorectal cancer in first-degree relatives, as well as alcohol and red meat consumption. These nutritional factors were assessed on average on a weekly basis during the last year, by a food frequency questionnaire previously validated in a subsample of French male adults (Kesse-Guyot et al. 2010).

### Cancer Data Collection

A follow-up study was organized to involve participants enrolled in the ARDCo program based on data concerning free medical care for cancer. In France, all cancers must be reported to the French National Health Insurance to provide full coverage of medical expenses, including treatment. Participants who applied to receive free medical care for colon or rectal cancer were identified each year during follow-up, which lasted from enrollment to 30 April 2014 (approximately 10 years).

### Statistical Analysis

***ARDCo analysis.*** We estimated associations between asbestos exposure and colon or rectal cancer among males in the ARDCo using separate Cox proportional hazard models with age as the main time variable, and with censoring on the date of diagnosis of colon or rectal cancer, date of death, or the end of follow-up (30 April 2014), whichever occurred first (Pairon et al. 2013). Two variables were used to characterize asbestos exposure: CEI modeled as a natural log–transformed continuous variable [expressed as ln(CEI + 1)] or categorized according to quartiles of exposure; and TSFE to asbestos, modeled as a time-varying continuous variable or as a categorical variable (0–20, > 20–40, ≥ 40–60, and ≥ 60 years) using 0–20 as reference. Proportionality assumptions of the Cox models were checked graphically (data not shown). All models were run separately for colon cancer and rectal cancer. We first ran separate univariate models of CEI and TSFE as continuous and categorical variables. In addition, we ran models that included both of the main exposures plus a categorical variable to adjust for smoking (never smoker, ex-smoker, current smoker, or missing), which was selected as a potential confounder *a priori*.

As crude results suggested the existence of an interaction between CEI and TSFE and incidence of colon cancer, we tested this hypothesis using a continuous model (CEI and TSFE) and also with a categorical model using CEI in quartiles and TSFE in two classes (< 40 and ≥ 40 years), adjusting both models for smoking. Accordingly, we provided stratified analyses on TSFE (< 40, ≥ 40 years) for colon cancer, but not for rectal cancer due to the insufficient number of cases of rectal cancer.

***ARDCo-Nut analysis.*** BMI was defined as a categorical variable using < 25, 25–< 30 and ≥ 30 classes. Exercise was defined by the duration of physical exercise per day in minutes, and dichotomized on the median value of the overall population (< 30, ≥ 30 min). Using specific algorithms, quantitative indices of daily consumption of red meat and alcohol were calculated using the food frequency questionnaire. Each variable was then dichotomized using the median value for the overall population (113.5 g/day and 146.4 mL/day, respectively). These variables, as well as other potential risk factors for colon and rectal cancer, were included in the full Cox models in the subsample of participants with the completed questionnaire on BMI, exercise, FAP, family history of colorectal cancer and nutritional factors. As for colon cancer, we also provided analyses stratified on TSFE (< 40, ≥ 40 years).

All models were based on complete case analyses except for smoking, for which a missing data category was also included in the analyses.

Statistical analysis was carried out using PROC FREQ, PROC MEANS, and PROC PHREG (version 9.3; SAS Institute, Inc, Cary, NC) and stset and stcox for survival analyses (release 13; Stata, College Station, TX). All statistical tests were two-sided, and statistical significance was defined as *p* < 0.05.

## Results

**Figure 1:**
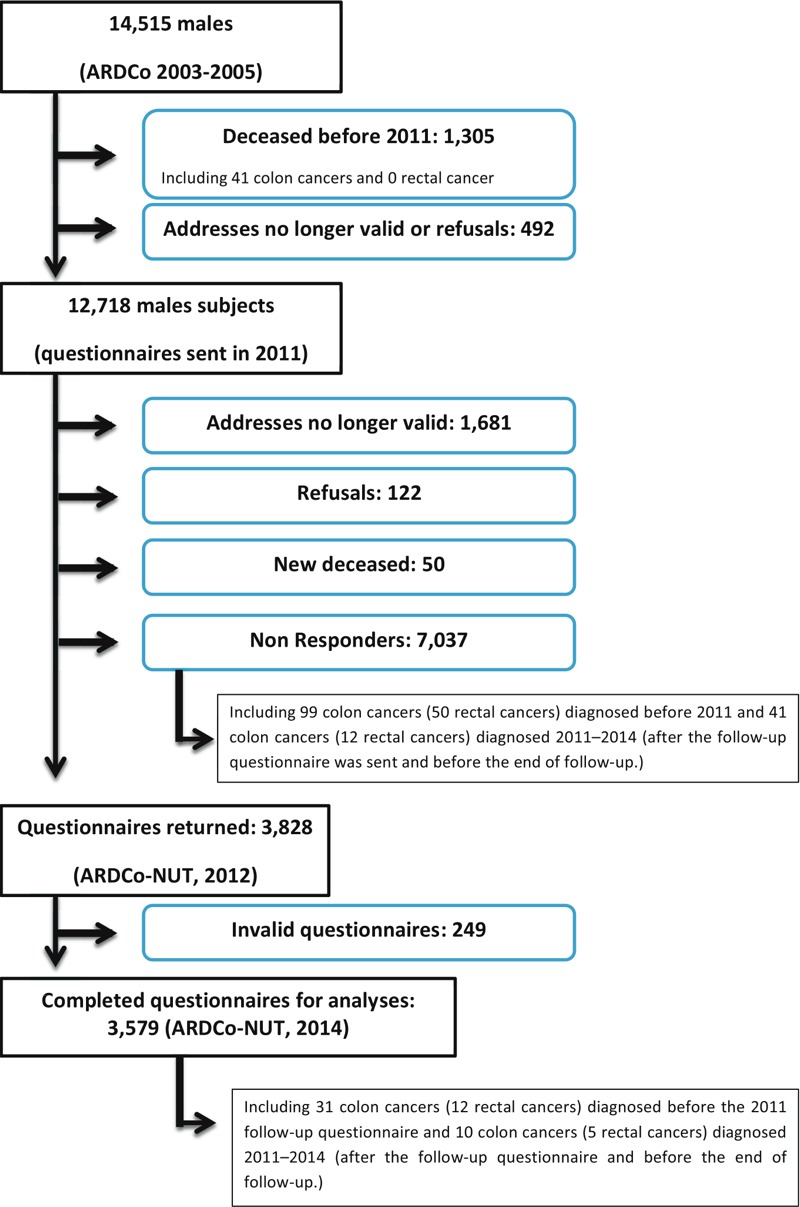
Flow-chart of the Asbestos-Related Diseases COhort (ARDCo) cohort and the ARDCo‑Nut subsample.

The ARDCo cohort comprised 14,515 men enrolled in 2003–2005, including 3,579 men who completed the ARDCo-Nut questionnaire in 2011 (Figure 1). In the overall cohort, 181 colon cancers and 62 rectal cancers were recorded by National Health Insurance between 2004 and 2014 (Table 1). The ARDCo-Nut subgroup included 41 colon cancer cases (31 diagnosed before and 10 diagnosed after completing the 2011 questionnaire) and 17 rectal cancer cases (12 diagnosed before and 5 diagnosed after completing the 2011 questionnaire). Overall duration of exposure was 30.9 years (SD 10.6), TSFE was 53.4 years (SD 7.5) and time since last exposures was 41.7 years (SD 14.8). No major differences were observed between the overall cohort and the ARDCo-Nut subgroup with the exception of smoking status, which was less likely to be missing in the ARDCo-Nut subgroup (14.6% versus 32.2%). Participants in the ARDCo-Nut study included 420 men (11.7%) with a family history of colorectal cancer, and 35 (1%) with a known family history of FAP (Table 2). Univariate analyses showed a significant inverse association between TSFE as a continuous variable and both cancers in the whole cohort (Table 3). Using TSFE as categorical variable, a positive significant association was observed between TSFE (20–< 40 relative to 0–< 20 years) and incidence of colon cancer [hazard ratio (HR) = 5.32; 95% confidence interval (CI): 2.21, 12.80] and rectal cancer (HR = 4.34; 95% CI: 1.10, 17.00), and a negative association was observed between TSFE ≥ 60 and colon cancer (HR = 0.36; 95% CI: 0.15, 0.92). No significant associations were observed with CEI. Multivariate analyses, including smoking status as confounding factor, confirmed the significant positive association between TSFE (20–< 40 years) as well as the negative association between TSFE ≥ 60 years and colon cancer. The positive association between TSFE (20–< 40 years) and rectal cancer was also confirmed. These analyses also indicated a significant positive association with CEI modeled as a continuous variable (HR = 1.14; 95% CI: 1.04, 1.26 for a 1-unit increase in lnCEI + 1), and suggested a positive relationship with CEI expressed as a categorical variable (HR = 1.17; 95% CI: 0.74, 1.85; HR = 1.55; 95% CI: 0.99, 2.42; HR = 1.54; 95% CI: 0.97, 2.45 relative to quartiles). No association with CEI was observed for rectal cancer alone. For colon cancer, there was a statistically significant interaction between TSFE and lnCEI when both were modeled as continuous variables adjusted for smoking (interaction *p* < 0.0001) (data not shown). When stratified by TSFE and adjusted for smoking, a positive association was observed between lnCEI + 1 and colon cancer among men with TSFE < 40 years (HR = 1.57; 95% CI: 1.25, 1.98 based on 1,166 total observations and 25 cases), but no association among men with TSFE ≥ 40 years (HR = 1.05; 95% CI: 0.95, 1.16 based on 13,349 total observations and 156 cases). Using CEI as categorical variable, a trend towards a positive relationship was also observed according to quartiles (HR = 4.96; 95% CI: 1.72, 14.27; HR = 3.13; 95% CI: 0.67, 14.47; HR = 9.12; 95% CI: 2.85, 29.80, trend test *p*-value = 0.0007) only among men with TSFE < 40 years. No significant association was observed for rectal cancer although a slight increase was observed for lnCEI + 1 among men with TSFE < 40 years (HR = 1.29; 95% CI: 0.91, 1.85).

**Table 1:** Study population characteristics of the overall ARDCo and the ARDCo‑Nut subsample (males only).

Characteristic	All participants (*n* = 14,515)	ARDCo-Nut (*n* = 3,579)
Age at baseline (years)^*a*^	63.2 ± 5.6	62.7 ± 5.2
< 60	2,825 (19.5%)	766 (21.4%)
60–74	11,235 (77.4%)	2,749 (76.8%)
≥ 75	455 (3.1%)	64 (1.8%)
Follow-up (years)
Median, min–max	10.2 (8.5–11.3)	10.2 (8.6–11.3)
Smoking status at baseline
Never smokers	2,960 (20.4%)	906 (25.3%)
Former smokers	6,005 (41.4%)	1,978 (55.3%)
Current smokers	857 (5.9%)	174 (4.9%)
Missing data	4,693 (32.3%)	521 (14.6%)
Duration of exposure to asbestos (years)^*a*^	30.9 ± 10.6	31.8 ± 10.3
Not exposed	656 (4.5%)	155 (4.3%)
< 20	2,162 (14.9%)	485 (13.5%)
20–29	2,679 (18.5%)	580 (16.2%)
30–39	5,925 (40.8%)	1,500 (41.9%)
≥ 40	3,093 (21.3%)	859 (24.0%)
Cumulative exposure index to asbestos, exposure-unit/years^*a*^	60.1 ± 99.1	58.2 ± 96.4
0–< 3	3,602 (24.8%)	822 (23.0%)
3–< 20	3,641 (25.1%)	622 (25.8%)
20–< 41	3,601 (24.8%)	934 (26.1%)
≥ 41	3,671 (25.3%)	901 (25.2%)
TSFE (years)^*a*^	53.4 ± 7.5	53.4 ± 7.0
Not exposed	656 (4.5%)	155 (4.3%)
< 40	496 (3.4%)	106 (3.0%)
40–49	3,207 (22.1%)	749 (20.9%)
50–59	7,648 (52.7%)	1,986 (55.5%)
≥ 60	2,508 (17.3%)	583 (16.3%)
Time since last exposure to asbestos (years)^*a*^	41.70 ± 14.8	41.23 ± 14.6
Not exposed	656 (4.5%)	155 (4.3%)
< 40	4,373 (30.1%)	1,148 (32.1%)
40–49	4,335 (29.9%)	1,089 (30.4%)
50–59	4,317 (29.7%)	1,001 (28.0%)
≥ 60	834 (5.8%)	186 (5.2%)
Colon cancer (yes)	181 (1.2%)	41 (1.1%)
Rectal cancer (yes)	62 (0.4%)	17 (0.5%)
Note: Min–max, minimum–maximum; *n*, overall number of participants by category; SD, standard deviation; TSFE, time since first exposure to asbestos. ^***a***^Data shown as mean ± SD.		

**Table 2:** Selected specific characteristics of the ARDCo‑Nut subsample in 2011 (males only, *n *= 3,579).

Characteristic	ARDCo-Nut
Smoking status
Never smokers	1,056 (29.5%)
Former smokers	2,351 (65.8%)
Current smokers	168 (4.7%)
Missing data	4
BMI (kg/m^2^)
< 25	1,139 (32.3%)
25 to < 30	1,787 (50.7%)
≥ 30	600 (17.0%)
Missing	53
Exercise^*a*^
< 30 min/day	1,097 (30.6%)
≥ 30 min/day	2,482 (69.4%)
Missing	0
History of familial adenomatous polyposis^*b*^	
No	3,544 (99.0%)
Yes	35 (1.0%)
Missing	165
Family history of colorectal cancer^*b*^
No	3,159 (88.3%)
Yes	420 (11.7%)
Missing	68
Red meat consumption (median, g/day)^*c*^	
< 113.5	1,741 (48.6%)
≥ 113.5	1,838 (51.4%)
Missing	0
Alcohol consumption (median, mL/day)^*c*^	
< 146.4	1,688 (47.2%)
≥ 146.4	1,891 (52.8%)
Missing	0
Note: BMI, body mass index. ^***a***^Defined at the time of the 2011 questionnaire as physical exercise per day such as walking, cycling, dichotomized on median value. ^***b***^Defined at the time of the 2011 questionnaire in first-degree relatives. ^***c***^Defined at the time of the 2011 questionnaire on a weekly basis for the last year and then dichotomized on median value.	

**Table 3:** Incidence of colon and rectal cancers according to asbestos exposure in the ARDCo (Cox models, *n* = 14,515).

Exposure	Colon cancer						Rectal cancer					
	Number		Univariate model		Multivariate model^*a*^		Number		Univariate model		Multivariate model^*a*^	
	*n*	C	HR (95% CI)	*p*-Value	HR (95% CI)	*p*-Value	*n*	C	HR (95% CI)	*p*-Value	HR (95% CI)	*p*-Value
Asbestos exposure CEI, (exposure-unit years)
Ln(CEI) + 1	14,515	181	1.04 (0.95, 1.13)	0.404	1.14 (1.04, 1.26)	0.007	14,515	62	0.89 (0.77, 1.03)	0.129	0.96 (0.82, 1.14)	0.662
0–< 3	3,559	43	Ref	0.695^*b*^	Ref	0.156^*b*^	3,582	20	Ref	0.159^*b*^	Ref	0.274^*b*^
3–< 20	3,600	41	0.94 (0.61, 1.44)	0.772	1.17 (0.74, 1.85)	0.500	3,622	19	0.93 (0.50, 1.75)	0.830	1.10 (0.56, 2.13)	0.772
20–< 41	3,550	51	1.19 (0.79, 1.78)	0.409	1.55 (0.99, 2.42)	0.052	3,593	8	0.18 (0.18, 0.92)	0.030	0.50 (0.21, 1.17)	0.109
≥ 41	3,625	46	1.10 (0.72, 1.67)	0.666	1.54 (0.97, 2.45)	0.067	3,656	15	0.39 (0.39, 1.49)	0.427	1.00 (0.48, 2.08)	0.997
TSFE (years)	14,515	181	0.98 (0.97, 0.99)	< 0.0001	0.98 (0.97, 0.99)	< 0.0001	14,515	62	0.98 (0.97, 0.99)	0.005	0.98 (0.97, 0.99)	0.030
0–< 20	663	7	Ref	< 0.0001^*b*^	Ref	< 0.0001^*b*^	663	3	Ref	0.0002^*b*^	Ref	0.0006^*b*^
20–< 40	478	18	5.32 (2.21, 12.80)	0.0002	4.67 (1.92, 11.46)	0.0007	482	7	4.34 (1.10, 17.00)	0.035	4.57 (1.14, 18.27)	0.030
40–< 60	10,729	141	1.33 (0.62, 2.85)	0.457	1.02 (0.45, 2.31)	0.972	10,813	49	1.05 (0.33, 3.36)	0.936	1.17 (0.34, 4.08)	0.802
≥ 60	2,464	15	0.36 (0.15, 0.92)	0.032	0.26 (0.10, 0.70)	0.0076	2,495	3	0.20 (0.04, 1.07)	0.060	0.23 (0.04, 1.32)	0.100
Note: C, overall number of incident colon or rectal cancer cases (update 30 April 2014); CEI, cumulative exposure index to asbestos; CI, confidence interval; HR, hazard ratio; *n*, overall numbers of participants by category; TSFE, time since first exposure (years). ^***a***^Models included smoking (nonsmokers as reference, former smokers, current smokers, and missing data as a category), CEI and TSFE separately, both as continuous variables or both as categorical variables. ^***b***^*p*-Value for trend test.												

The same analyses were then performed on the ARDCo-Nut subsample (Table 4). In multivariate analyses, no association was observed between CEI and asbestos and between colon or rectal cancer incidence. When stratified by TSFE, a positive association was observed between lnCEI + 1 and colon cancer among men with TSFE < 40 years (HR = 1.94; 95% CI: 1.23, 3.07 based on 264 total observations and 5 cases, adjusted for family history of FAP and colon cancer), but no association was observed among men with TSFE ≥ 40 years (HR = 0.95; 95% CI: 0.77, 1.17 based on 3,315 total observations and 36 cases, adjusted for smoking, BMI, exercise, family history of FAP and colon cancer, and red meat and alcohol consumption).

**Table 4:** Incidence of colon and rectal cancers according to asbestos exposure in the ARDCo‑Nut sample (Cox models, *n* = 3,579).

Exposure	Colon cancer						Rectal cancer					
	Number		Univariate model		Final model^*a*^		Number		Univariate model		Final model	
	*n*	C	HR (95% CI)	*p*-Value	HR (95% CI)	*p*-Value	*n*	C	HR (95% CI)	*p*-Value	HR (95% CI)	*p*-Value
CEI (exposure-unit years)
Ln(CEI) + 1	3,538	41	1.01 (0.84, 1.21)	0.946	1.07 (0.87, 1.32)	0.522	3,562	17	0.89 (0.67, 1.19)	0.443	0.90 (0.65, 1.24)	0.512
0–< 3	811	11	Ref	0.868^*b*^	Ref	0.912^*b*^	818	4	Ref	0.270^*b*^	NA	NA
3–< 20	912	10	0.83 (0.35, 1.96)	0.674	0.84 (0.33, 2.11)	0.708	914	8	1.78 (0.54, 5.92)	0.345	NA	NA
20–< 41	922	12	1.00 (0.44, 2.27)	0.997	1.07 (0.44, 2.57)	0.885	932	2	0.45 (0.08, 2.48)	0.361	NA	NA
≥ 41	893	8	0.72 (0.29, 1.79)	0.479	0.80 (0.30, 2.14)	0.662	898	3	0.74 (0.17, 3.32)	0.696	NA	NA
TSFE (continuous years)	3,538	41	0.99 (0.97, 1.04)	0.135	0.98 (0.96, 1.00)	0.074	3,562	17	0.99 (0.96, 1.03)	0.705	0.99 (0.96, 1.03)	0.774
0–20	155	1	Ref	0.0031^*b*^	Ref	0.004^*b*^	156	0	NA	NA	NA	NA
20–40	104	4	11.48 (1.24, 105.75)	0.031	11.45 (1.21, 108.44)	0.034	107	1	NA	NA	NA	NA
40–60	2,705	32	2.50 (0.33, 18.71)	0.372	2.46 (0.30, 20.19)	0.402	2,725	12	NA	NA	NA	NA
≥ 60	574	4	0.78 (0.09, 7.01)	0.821	0.69 (0.07, 7.03)	0.753	574	4	NA	NA	NA	NA
Note: C, overall number of incident colon or rectal cancer cases (update 30 April 2014); CEI, cumulative exposure index to asbestos; CI, confidence interval; HR, hazard ratio; *n*, overall numbers of participants by category; NA, not applicable; TSFE, time since first exposure (years). ^***a***^Model adjusted on smoking, BMI, physical exercise, familial history of FAP or colorectal cancer in first-degree relatives, daily red meat and alcohol consumption. Models included separately both CEI and TSFE as categorical variables, or as continuous variables. ^***b***^*p*-Value for trend test.												

No association was observed between asbestos exposure, according to the two categories of TSFE, and rectal cancer, although a slight increase in rectal cancer was observed with CEI to asbestos in the lower TSFE stratum (HR = 1.30; 95% CI: 0.91, 1.85), but not in the upper stratum (HR = 0.90; 95% CI: 0.75, 1.07). However, these results are difficult to interpret in view of the small number of cases in this analysis.

## Discussion

This study, based on a large cohort with a follow-up of more than 10 years, supports a positive association between cumulative exposure to asbestos and the incidence of colon cancer. In addition, a significant positive association was observed for TSFE between 20 and 40 years but a significant negative association was observed for TSFE ≥ 60 years. It is noteworthy that the positive association between CEI and colon cancer was only observed after adjusting for TSFE and smoking, and was only evident among men with TSFE < 40 years old. Rectal cancer was also positively and significantly associated with TSFE 20–< 40 years, but not with CEI, after adjusting for smoking. However, these findings were based on small numbers of cases. To our knowledge, this study, presenting results adjusted for smoking, family history of colon cancer and FAP, exercise, BMI, and red meat and alcohol consumption is one of the largest incidence studies on this topic.

Numerous studies, mostly based on mortality cohorts, have reported an association between colorectal cancer and asbestos exposure (Gamble 1994). However, Homa et al. (1994) who reviewed 20 mortality studies noted that no clear relationship could be demonstrated between asbestos exposure and colorectal cancer death on the basis of these studies. Several mortality studies have subsequently reported a positive association for colon and rectal cancer and asbestos exposure (Berry et al. 2000; Germani et al. 1999; Kang et al. 1997; Lin et al. 2014). For instance, Berry et al. (2000) reported an excess of colon cancer mortality in a cohort of 5,100 asbestos factory workers, particularly among insulators [standardized mortality ratio (SMR) = 1.8 (1.2–2.7)], who were followed between 1933 and 1980. However, over the same period, several cohort studies failed to demonstrate an association between asbestos exposure and colorectal cancer death (Battista et al. 1999; Dement et al. 1994; Ferrante et al. 2007; Giaroli et al. 1994; Hein et al. 2007; Levin et al. 1998; Loomis et al. 2009; Rösler et al. 1994; Wang et al. 2013a).

Only a few studies, prior to the present study, have reported incidence data. Aliyu et al. (2005) compared the incidence of colorectal cancer in the United States between 1,839 heavy smokers who were exposed to asbestos and 7,924 heavy smokers who were not exposed to asbestos; they estimated a relative risk (RR) of 1.36 (95% CI: 0.96, 1.93). Clin et al. (2011) reported a significant trend *p*-value for the association between tertiles of asbestos exposure and the incidence of colorectal cancer (25 cases) among 2,024 former textile and friction material industry workers in France who were heavily exposed to asbestos (Clin et al. 2011). In contrast, Koskinen et al. (2003) observed no significant associations with colon cancer or rectal cancer incidence rates among 23,285 Finnish men (67 and 60 cases, respectively) and 939 women (3 and 1 cases, respectively), who were eligible for a screening program of asbestos-related diseases among workers with possible occupational exposure. Consistent with these discordant results, the Institute of Medicine (IOM) concluded, in 2006, that evidence for a causal association between asbestos and colorectal cancer was “suggestive but not sufficient” (IOM 2006), while a more recent review concluded that the evidence was “limited” (IARC 2012).

One of the unresolved issues concerns the possible existence of a dose–response relationship between asbestos exposure and colon cancer risk. McDonald et al. (1980) first observed a significant trend between high level of asbestos exposure and colorectal cancer deaths in a large-scale mortality study that included 10,939 men. However, only rare studies have reported a clear dose–response relationship between asbestos exposure and colorectal cancer. In a cohort study based on 1,929 asbestos workers, who were mainly exposed to chrysotile and for whom dust measurements were available between 1956 and 1977, Albin et al. (1990) reported a significant slope of 1.6% (0.2%–3.1%) per fiber year/mL for cancer mortality. Recently, in chrysotile asbestos miners, Wang et al. (2013b) reported a significant trend for gastrointestinal cancer death and cumulative exposure to asbestos, with a significant excess risk for an exposure > 100 fiber years/mL. To our knowledge, only two studies have reported some evidence of a dose–response relationship between asbestos exposure and colon cancer incidence. Clin et al. (2011) reported a significant trend for tertiles of average exposure intensity (HR = 3.86; 95% CI: 0.47, 31.9 and HR = 7.20; 95% CI: 0.91, 56.7 for the second and third tertiles compared with the first tertile, respectively, trend *p*-value 0.02), but this analysis was based on only 25 cases (1 case in the lowest tertile), and they found no association with the cumulative exposure index to asbestos (Clin et al. 2011). A recently published, large-scale (58,279 men) prospective study using a job exposure matrix to assess asbestos exposure reported a significant association with colon cancer incidence for men in the highest tertile of heavy asbestos exposure duration (median 30 years) compared with men who were never highly exposed (HR = 2.19; 95% CI: 1.04, 4.19), but HRs for the first and second tertiles of heavy exposure were < 1 (Offermans et al. 2014). No significant trend was observed with duration of exposure or cumulative exposure when considering all participants and not only heavily exposed participants.

However, most of the studies cited above did not take into account other risk factors for colorectal cancer, particularly cohort mortality studies except for one study that presented results adjusted for smoking status (Wang et al. 2013b). Smoking is the adjustment factor most frequently used in incidence studies, but other factors such as family history of digestive cancer, BMI or alcohol consumption were not taken into account, except in the study by Offermans et al. (2014). Case–control studies were obviously more frequently adjusted for these factors, but their results were also often discordant as both positive (Goldberg et al. 2001) and negative studies (Fang et al. 2011; Garabrant et al. 1992) have been published.

Our study does not provide any clear evidence of an association with rectal cancer, but our findings should be interpreted cautiously in view of the smaller number of cases of rectal cancer compared to colon cancer (62 vs. 181, respectively, in the overall cohort). However, a nonsignificant negative association was observed with lnCEI, in contrast with the significant positive association observed for colon cancer.

Most of the studies discussed above did not distinguish between these two cancers making it impossible to compare our results with those of previous studies. As in the present study, some mortality cohorts have reported a significant association between asbestos exposure and colon cancer but not rectal cancer (Berry et al. 2000; Jakobsson et al. 1994). As already mentioned, Offermans et al. (2014) found a suggestive relationship for colon cancer incidence, but also for rectal cancer with a positive significant association in ever highly exposed participants (HR = 2.15; 95% CI: 1.23, 3.77).

An association between asbestos exposure and colon cancer is also supported by other evidence. Ingestion of chrysotile or crocidolite in rats induced aberrant crypt foci, considered to be a premalignant step of colon cancer (Corpet et al. 1993). Asbestos fibers have also been shown to diffuse into digestive organs after inhalation or ingestion (Masse et al. 1980). Moreover, in humans, it is estimated that about one twentieth of inhaled asbestos is subsequently ingested (Schneiderman 1974). Kjaerheim et al. (2005) reported an excess of colon cancer (OR = 1.6; 95% CI: 1.0, 2.5) with a latency of 20 years, in lighthouse keepers exposed to an average of 7.1 × 10^10^ fibers/L of asbestos, but Browne et al. (2005) did not find any association between asbestos in drinking water and colon cancer. Overall, these studies suggested that asbestos exposure may interact with the colon carcinogenesis, although the results are inconclusive.

Some limitations of our study need to be discussed. First of all, the ARDCo participants are derived from a selected population, as only male volunteers were included in the survey. Exposure assessment was also retrospective, with no atmospheric measurements, which could also modify the relationships between exposure parameters and colon cancer incidence. However, our previous publications have demonstrated a very strong relationship between CEI and pleural plaques, supporting satisfactory assessment of asbestos exposure (Paris et al. 2009). The ARDCo-Nut study population was not very large, and some analyses were limited by the small number of colon cancers. Moreover, as ARDCo-Nut questionnaires were sent several years after enrollment, 130 of the 181 men with colon cancer were diagnosed before receiving the follow-up questionnaire, including 99 who did not respond to the follow-up questionnaire, and 31 who were diagnosed with colon cancer before they completed the follow-up survey. Only 10 of the 51 men who were diagnosed with colon cancer after the follow-up survey completed the questionnaire. Similar features were observed for rectal cancers, as 50 men (out of 62) were diagnosed before the follow-up questionnaire (12 responders), and among the remaining 12 cases, only 5 were responders. It is therefore difficult to predict the potential influence of self-selection, loss to follow-up including loss due to death and non-response, on our estimates.

The role of TSFE also needs to be considered in our study. At first sight, the overall significant inverse relationship between TSFE and colon cancer may appear to be unusual. We estimated a significantly higher relative risk of colon cancer for men with TSFE between 20 and 40 years compared with TSFE < 20 years, and a significantly lower relative risk for men with ≥ 60 years TSFE. However, with a mean TSFE of 53.4 (13–86) years in the overall cohort, the present study is based on very long follow-up. Data on long latency and asbestos-related cancers are rare, except for mesothelioma. Pira et al. (2005) reported a significant association between TSFE and both mesothelioma and lung cancer mortality, with a slight decrease of SMR for a TSFE ≥ 35 years relative to a TSFE between 25 and 35 years. However, in this study, no significant relationship was observed between TSFE and colorectal cancer mortality. In conclusion, our findings are relatively consistent with the literature, but the role of a TSFE < 20 years could not be tested, as only 13 exposed participants had a TSFE < 20 years.

Another possible limitation concerns the confirmation of the diagnosis of colon cancer. We compared data from French National Health Insurance with data from cancer registries available in four small areas of the study covering 27% (*n* = 4,348) of the overall ARDCo cohort. Of the 44 cases recorded in the French National Health Insurance database, 39 had a confirmed diagnosis of colorectal cancer in cancer registries, while the remaining 5 cases were not included in these registries. This finding indicates that our data can be considered to be fairly complete. Discrepancies between the two databases may be explained by errors in database linking, and delayed registration in cancer registries (an average of 2 years) compared to the National Health Insurance database.

As previously discussed, this study comprised several adjustments for *a priori* risk factors for colon cancer namely BMI, exercise, family history of FAP or colorectal cancer, and red meat and alcohol consumption. However, we cannot rule out the presence of residual confounding. In particular, we failed to reproduce significant associations with most of these factors, which can probably be explained by the relatively small number of cases of colon cancer (*n* = 41) in the ARDCo-Nut subsample.

## Conclusion

We estimated a significant positive association between cumulative exposure to asbestos and the incidence of colon cancer in a large prospective cohort. This association was only evident among men with a TSFE < 40 years. The association was also observed after adjustment for BMI, exercise, and family history of FAP in a subsample of the initial cohort. In the light of previous studies, and certain experimental data, although sparse, this study supports an association between asbestos exposure and colon cancer. Our study did not provide any clear evidence of an association with rectal cancer, but these findings should be interpreted cautiously in view of the insufficient number of cases.
